# Computed Tomographic Imaging of Subchondral Fatigue Cracks in the Distal End of the Third Metacarpal Bone in the Thoroughbred Racehorse Can Predict Crack Micromotion in an Ex-Vivo Model

**DOI:** 10.1371/journal.pone.0101230

**Published:** 2014-07-31

**Authors:** Marie-Soleil Dubois, Samantha Morello, Kelsey Rayment, Mark D. Markel, Ray Vanderby, Vicki L. Kalscheur, Zhengling Hao, Ronald P. McCabe, Patricia Marquis, Peter Muir

**Affiliations:** 1 Comparative Orthopaedic Research Laboratory, School of Veterinary Medicine, University of Wisconsin-Madison, Madison, Wisconsin, United States of America; 2 Department of Orthopedics & Rehabilitation, School of Medicine & Public Health, University of Wisconsin-Madison, Madison, Wisconsin, United States of America; 3 Gulfstream Park, Hallandale Beach, Florida, United States of America; University of California Davis, United States of America

## Abstract

Articular stress fracture arising from the distal end of the third metacarpal bone (MC3) is a common serious injury in Thoroughbred racehorses. Currently, there is no method for predicting fracture risk clinically. We describe an ex-vivo biomechanical model in which we measured subchondral crack micromotion under compressive loading that modeled high speed running. Using this model, we determined the relationship between subchondral crack dimensions measured using computed tomography (CT) and crack micromotion. Thoracic limbs from 40 Thoroughbred racehorses that had sustained a catastrophic injury were studied. Limbs were radiographed and examined using CT. Parasagittal subchondral fatigue crack dimensions were measured on CT images using image analysis software. MC3 bones with fatigue cracks were tested using five cycles of compressive loading at -7,500N (38 condyles, 18 horses). Crack motion was recorded using an extensometer. Mechanical testing was validated using bones with 3 mm and 5 mm deep parasagittal subchondral slots that modeled naturally occurring fatigue cracks. After testing, subchondral crack density was determined histologically. Creation of parasagittal subchondral slots induced significant micromotion during loading (*p*<0.001). In our biomechanical model, we found a significant positive correlation between extensometer micromotion and parasagittal crack area derived from reconstructed CT images (S_R_ = 0.32, *p*<0.05). Correlations with transverse and frontal plane crack lengths were not significant. Histologic fatigue damage was not significantly correlated with crack dimensions determined by CT or extensometer micromotion. Bones with parasagittal crack area measurements above 30 mm^2^ may have a high risk of crack propagation and condylar fracture in vivo because of crack micromotion. In conclusion, our results suggest that CT could be used to quantify subchondral fatigue crack dimensions in racing Thoroughbred horses in-vivo to assess risk of condylar fracture. Horses with parasagittal crack arrays that exceed 30 mm^2^ may have a high risk for development of condylar fracture.

## Introduction

Parasagittal fracture of the condyles of the third metacarpal/metatarsal bone (MC3/MT3), or condylar fracture, is common in Thoroughbred and Standardbred racehorses and is often identified as part of a syndrome of fetlock breakdown injury. Some of these injuries are catastrophic and require euthanasia of the horse [1], [2]. In horses where surgical treatment is indicated, prognosis for return to athletic activity is not always favorable [2]–[4]. Risk of catastrophic injury may be as high as 2.17%, or 1 in every 46 race starts at some National Hunt racecourses in the United Kingdom [5]. Metacarpal fracture is the second most common cause of fatalities arising from a fracture in United States [6]. In the United Kingdom, condylar fractures are the most common type of fracture associated with racing and the most common reason for euthanasia of Thoroughbred racehorses with fracture [7].

Over the last 30 years, various etiologies for condylar fracture have been proposed. These theories fall into three main categories: first, accidental injury from mechanical overload [8]; second, pathological fracture associated with traumatic osteochondrosis [9]–[10]; and third, failure of functional adaptation and development of stress fracture [11]–[14]. Functional adaptation is a process by which bone remodels in response to mechanical loading. Bones are typically adapted to normal loads, but may be poorly designed to resist propagation of macroscopic fatigue cracks [15]. Although research data are primarily associative, it is now widely accepted that condylar fractures are site-specific articular stress fractures that essentially represent failure of functional adaptation to protect the subchondral bone plate of affected joints from cumulative fatigue injury [11]–[16]. Functional strains normally range from −1,500 to −3,000 microstrain in cursorial mammals [17]. However, in-vivo strains of more than −5,000 microstrain have been recorded in the MC3 mid-diaphysis of Thoroughbreds during galloping [18].

Functional adaptation is readily detectable by 4 months of race training [19]. Contact stresses on the palmar or plantar regions of the distal end of the MC3/MT3 bones from the proximal sesamoid bones are more than twice the stresses imposed on the dorsal region at the canter; this is a result of more load being shifted to the suspensory apparatus during increased fetlock joint extension [20]. As training increases, adaptation in the subchondral plate leads to sclerosis of the trabecular bone in the palmar/plantar aspect of the condyles, endochondral ossification of the joint surface, and advancement of the tidemark to the articular surface [11], [14]. These changes are associated with site-specific microdamage accumulation in calcified cartilage and the underlying subchondral bone of the parasagittal condylar grooves [11], [12], [14]. Microcrack initiation occurs in the calcified cartilage layer [14] and stimulates a targeted remodeling response that results in the formation of resorption spaces containing activated osteoclasts in the damaged bone [14]. This reparative response is associated with an increase in bone porosity, and may make horses more vulnerable to stress fracture if athletic activity is ongoing [14]. Accumulation and coalescence of these microcracks leads to development of macroscopic crack arrays in the subchondral bone of the condylar grooves [11], [12], [14]. Crack propagation through porous bone compromises the overlying cortical shell at the distal end of the MC3/MT3 bone [15], [21]. Once the cortical shell of the distal end of the MC3/MT3 bone is mechanically compromised, crack propagation proximally along trabecular planes can easily develop, thereby rendering the horse at high risk of developing condylar stress fracture [15], [21].

Currently, there are differing opinions in the field regarding the etiology of fetlock breakdown injury in the racing Thoroughbred. The causative mechanism is likely complex and involves all of the joint structures, the third metacarpal or metatarsal, the proximal sesamoids, and the proximal phalanx. Very little is known about the mechanical stability of the fatigue cracks in the subchondral bone plate that precede development of a condylar stress fracture. It is likely that condylar stress fracture is preceded by cracks in the subchondral plate that become sufficiently severe to permit development of micromotion at the high joint loads associated with training or racing. Therefore, knowledge of the relationship between crack dimensions determined by cross-sectional imaging and crack micromotion at high load may facilitate early identification of horses that are at high risk of condylar stress fracture. Ultimately, this knowledge could help reduce the incidence of serious or catastrophic injuries at the racetrack.

Multiple imaging methods relevant to the distal limb are available to the equine clinician. Radiography is inferior to computed tomography (CT) and magnetic resonance imaging (MRI) for identification of pathologic features in subchondral bone and articular cartilage, such as fatigue cracks in the subchondral bone plate [22], [23]. In addition to increased sensitivity for identifying pathologic changes in the articular surface of the fetlock joint, CT allows for multi-planar analysis of cross-sectional images of bone, facilitating a more accurate determination of size or extent of specific structures, such as subchondral fatigue cracks.

The objective of the present study was to develop an ex-vivo biomechanical model in which subchondral crack micromotion at high loads could be measured in distal MC3. We then used this model to determine the relationship between subchondral crack dimensions measured using CT and crack micromotion. We hypothesized that subchondral crack micromotion would be commonly detectable in the distal end of the MC3 bone in Thoroughbred racehorses at joint loads that model racing activity. Such a result would suggest that athletic Thoroughbred horses commonly train and race with incipient condylar stress fracture and are vulnerable to fracture propagation during athletic activity. A secondary objective was to study detection of fetlock joint abnormalities by CT imaging.

## Materials and Methods

### Thoroughbred racehorses

**Figure 1:**
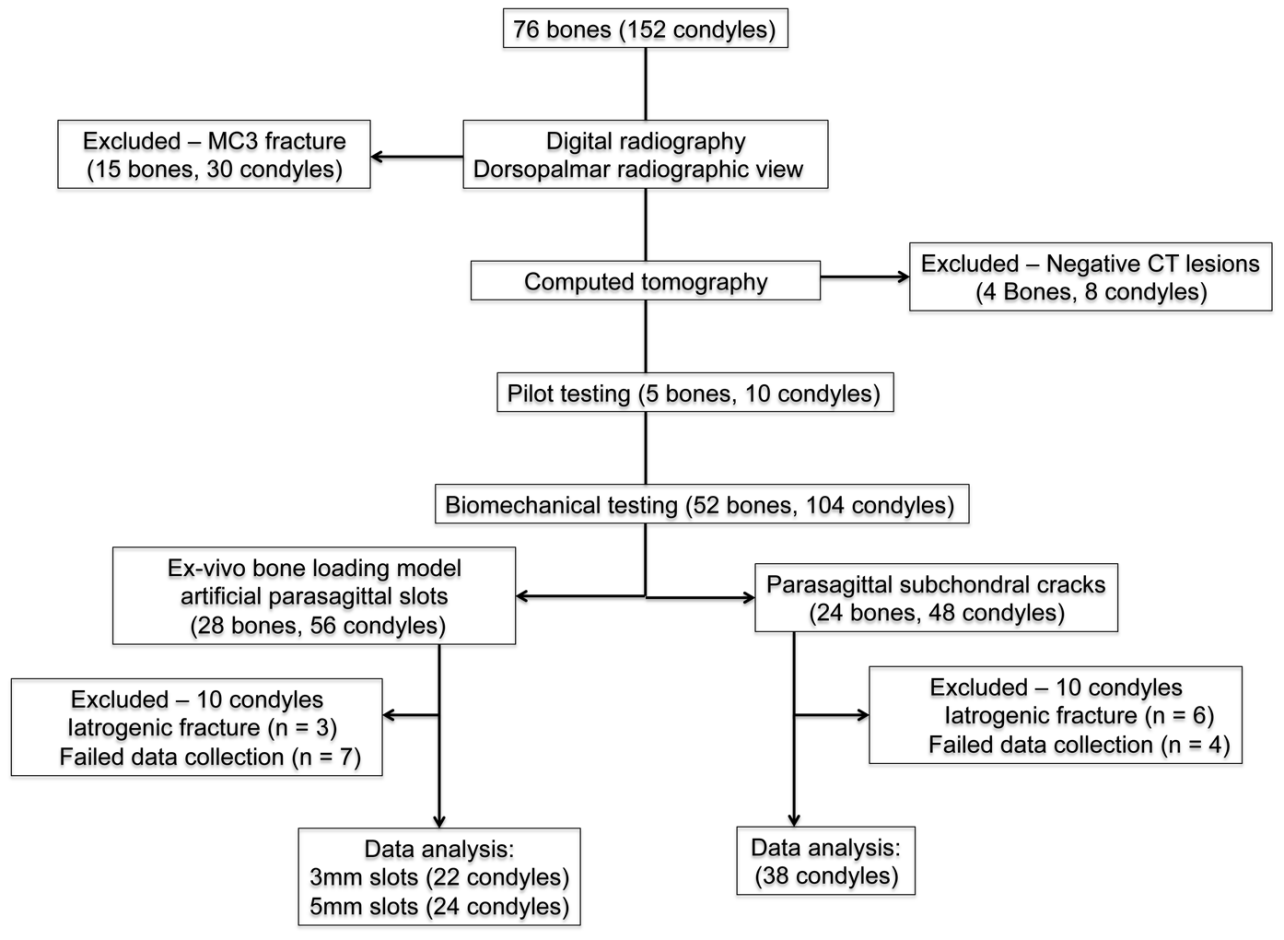
Flow diagram for limb inclusion and exclusion. Of the 152 condyles from 76 limbs horses initially screened, 38 condyles with naturally occurring subchondral fatigue cracks were ultimately studied in detail.

Thirty-six pairs of entire distal forelimbs and 4 individual limbs from 40 Thoroughbred racehorses that died or were euthanatized for reasons unrelated to the present study were given for use in this work (**Fig. 1**). Horses were euthanatized humanely by a veterinarian at the racetrack using an intravenous anesthetic overdose. Euthanasia was performed for clinical reasons because of serious injury during racing. Only thoracic limbs were used for this study. Condylar fractures most commonly affect the thoracic limbs [2], [3], [24], [25]. Limbs were transected at the level of the carpus, sealed in plastic bags, and stored at -20C until needed. The age, gender, and racing history were collected from the Jockey Club Information Systems, Inc. Thoroughbred racehorse database (www.equineline.com) for horses with parasagittal subchondral cracks that were tested mechanically.

### Digital radiography (DR)

DR radiographs of the metacarpophalangeal joint of each limb specimen were made using a single dorsopalmar radiographic view (70 kVp, 0.08 Ms, EDR3-MFA, Sound-Eklin, Carlsbad, CA) to determine whether fractures of the MC3 bone, proximal sesamoid bones or proximal phalanx were present. Limbs were excluded if a fracture of the MC3 bone was present.

### Computed tomography (CT)

**Figure 2:**
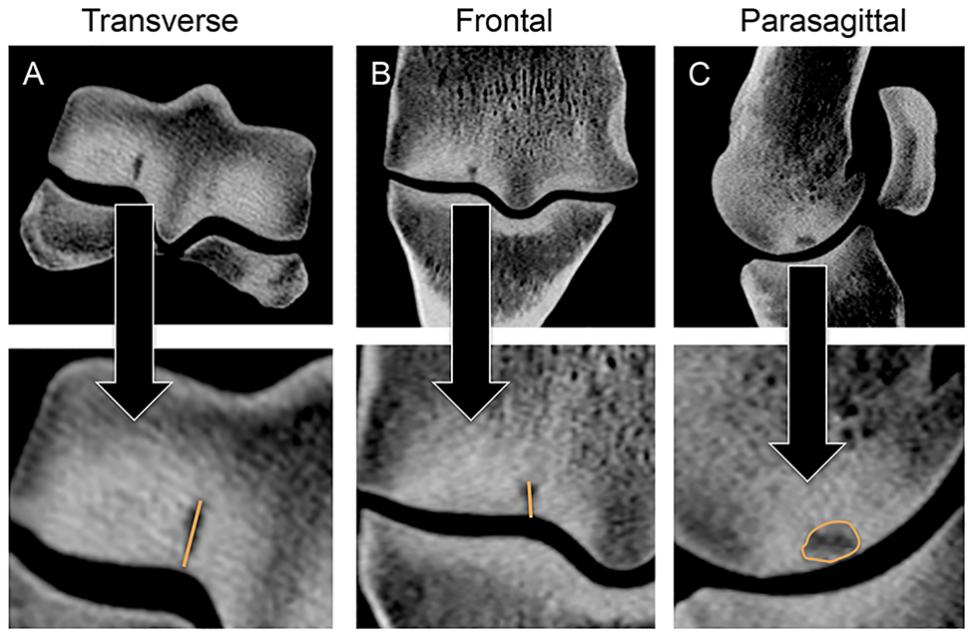
Subchondral fatigue crack dimensions were measured in transverse (**A**) and reconstructed frontal plane (**B**) computed tomography (CT) images. In addition, the area of the crack array was measured in the parasagittal plane (**C**).

CT imaging was performed at high-resolution using 0.625 mm contiguous slices (64 slices GE Discovery CT750 HD, GE Healthcare Technologies, Waukesha, WI, USA). CT images were reconstructed in the sagittal, transverse, and frontal planes using a 64-bit Dicom viewer (OsiriX, Pixmeo SARL, Bernex, Switzerland). CT images were examined for evidence of pathological changes, including the presence of subchondral sclerosis, fractures, defects in the joint surface, and parasagittal fatigue cracks within the condylar grooves. The presence of palmar osteochondral disease (POD), initially referred to in the literature as traumatic osteochondrosis [26], [27], was defined by the presence of subchondral bone lysis in the palmar condyle area [22]. The dimensions of subchondral cracks were measured radiographically on transverse and reconstructed frontal plane CT images (**Fig. 2**). In addition, crack area was measured in the parasagittal plane using image analysis (Image J, NIH) (**Fig. 2**). Using commercially available software (Mimics 13.1 Materialise, Ann Arbor, Michigan), the distal end of each MC3 bone with a parasagittal condylar groove lesion was segmented manually. A 3D volumetric model of the subchondral crack lesion was then created from the segmented images on a voxel-by-voxel basis to yield measurements of subchondral crack volume and the surface area of the subchondral crack volume.

### Visual examination and photography

After CT imaging, all soft tissue was removed from each MC3 bone. The shaft was cut with a band saw in the mid-diaphysis, 12 cm proximal to the dorsal articular margin of the distal end of the bone. The second and fourth metacarpal bones were removed. The articular surface at the distal end of the bone was photographed and the articular cartilage and remaining soft tissues were then digested using 0.15–0.2 M sodium hydroxide at 37°C. The solution was changed as needed every 1 to 3 days. Once the soft tissues were removed, bones were fixed in 70% ethanol and additional photographs of the distal articular surface were made. Severity of the parasagittal crack arrays was graded using a visual analogue scale as previously described [12]. The articular surface was also evaluated for any other pathology before and after digestion. The presence of POD was considered positive if there was discoloration of the cartilage or subchondral bone of the palmar condyle, ulceration/collapse of the cartilage, or presence of subchondral defect in the palmar region of the condyle [26], [27]. Severity of POD was also graded using a visual analogue scale [12].

### Ex-vivo bone loading model

To study subchondral crack micromotion at high loads associated with athletic performance, we developed a non-destructive MC3 bone-loading model. An isolated single bone model was chosen for this work because of the high compressive load used for mechanical testing. Each bone was potted in an aluminum cylinder that was 10 cm long using epoxy (Evercoat 100156 Lite Weight Autobody Filler, Fiber Glass-Evercoat, Cincinnati, OH, USA). An 8 mm hole was drilled in the proximal end of the potted bone and the surrounding cylinder approximately 2 cm from the end of the cylinder, perpendicular to the long axis of the bone and the joint surface, and a stainless steel pin was inserted for additional stability.

**Figure 3:**
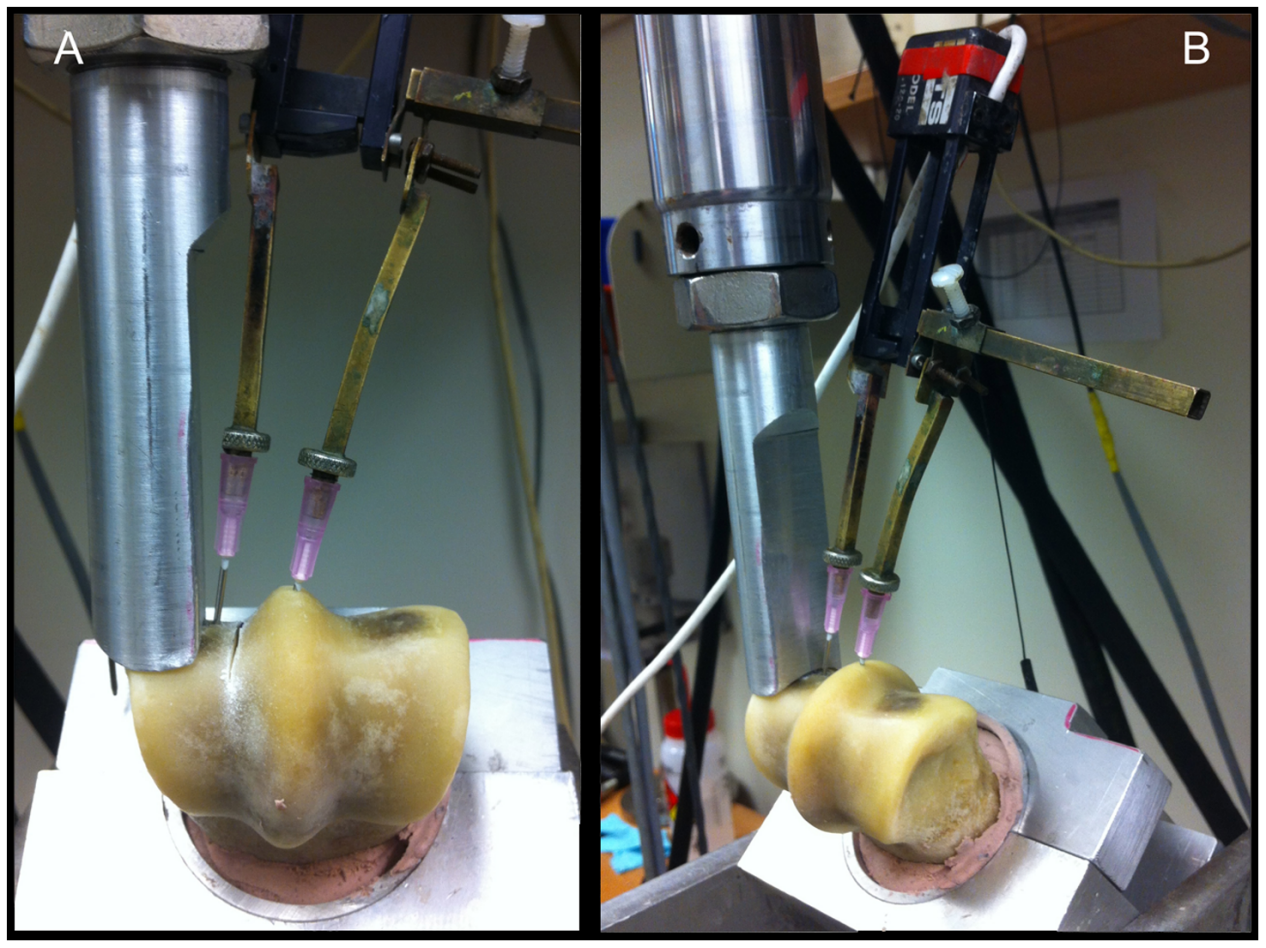
Photograph illustrating the custom-made jig that secures the distal end of the MC3 bone to the platen of a materials testing machine (MTS 858, Minneapolis, MN). (**A**) Drill holes 1.25 mm in diameter were made on the lateral and medial side of the artificial slot or crack array in the parasagittal condylar groove. (B) Hypodermic needles (18 g) were then placed in the drill holes and attached to an extensometer to measure motion across the slot or crack. The actuator consisted of a metal rod that was contoured to conform to the curved surface of the condyle.

Bone specimens without detectable CT lesions in the condylar grooves were used to validate the biomechanical model. Holes, 1.25 mm in diameter, were drilled at the distal palmar aspect of the sagittal ridge and 1 mm abaxial to the condylar groove in the same oblique frontal plate to span the region-of-interest. The potted bone specimen was then positioned in a custom jig (**Fig. 3**). The bones were oriented obliquely in the jig with the palmarodistal aspect facing up in order to mimic compressive loading by the proximal sesamoid bones from weight-bearing. Hypodermic needles (18 gauge) were placed in the drill holes and connected to an extensometer (MTS Systems Corp., Model 632-120-20, Eden Prairie, MN). Lateral and medial condyles of each bone were tested separately. As the bones were not tested destructively each specimen acted as it's own control. Each intact condyle was tested once, in order to obtain a baseline extensometer measurement before creation of an artificial subchondral slot to model an in-vivo fatigue crack.

Crack micromotion was measured using a materials testing system (modified MTS Bionics 858 hydraulic test machine; MTS Systems Corp, Eden Prairie, Minneapolis, MN) under load control. Before the mechanical test, each bone was pre-loaded at 100 N. Five cycles of compressive load of 7,500 N were then applied to the condyle abaxial to the extensometer at 0.1 Hz. This load was selected based on biomechanical modeling of fetlock joint loading [20]. Data were collected at 30 Hz for all channels (load, displacement and extensometer motion) and stored on a computer in a data file for future analysis. Mean micromotion for each test was determined by peak-to-peak measurements for the five load cycles. It has been previously estimated that the peak compressive load on the palmar surface of the distal end of the MC3 bone exerted by each proximal sesamoid bone on its respective condyle is at least 7,500 N for a 500 kg horse at the canter, giving rise to a peak stress of 23.9 MNm^−2^ [20].

After an initial baseline testing, an artificial slot was created in the palmar region of the condylar groove using a high-speed rotary tool with a circular diamond wheel, 0.6 mm thick (Dremel 545, Racine, WI) to model a naturally occurring parasagittal array of fatigue cracks. Lateral and medial condyles were randomly assigned to slots that were either 3 mm or 5 mm deep and approximately 18 mm long in a dorsopalmar direction. Mechanical testing was then repeated.

### Mechanical testing of parasagittal subchondral fatigue cracks

Bone specimens that were found to have macroscopic parasagittal crack arrays in the condylar groove after cartilage digestion and corresponding lesions detectable on CT cross-sectional images were also tested biomechanically. In order to separate pre-existing fatigue cracking from artifactual damage induced by mechanical testing, a bulk-staining method was used [28]. The entire distal end of the MC3 bone was bulk-stained in 1% basic fuchsin for three days, changing the solution daily. The bones were then placed in 100% ethyl alcohol for 20 minutes before being incrementally rehydrated and then placed in 0.9% saline. The bones were mounted and tested as described above. Movement during loading of the condyle was detected by placement of the extensometer across the array of cracks in the condylar groove. Lateral and medial condyles were again tested separately, but testing was not repeated.

### Bone morphometry

After mechanical testing, 1.1 mm thick oblique frontal sections of the distal end of each MC3 bone were prepared using a diamond saw. Sections were cut in a proximodorsal distopalmar plane to pass through the palmar crack array in the condylar groove [12]. Microradiographs (Faxitron, Wheeling, IL, USA) of each section were made for comparison with equivalent frontal plane CT images.

Bone sections were examined for condylar groove cracking [14]. Subchondral cracks that were stained with basic fuchsin were counted within the condylar groove region and normalized to the length of the regional bone boundary to give a crack boundary density (N.Cr/B.Bd, #/mm) by a single observer (KR) [14]. Sections were also examined for unstained artifactual subchondral cracks associated with mechanical testing and bone sectioning. In addition, stained subchondral cracks were examined for evidence of crack extension during mechanical testing.

### Statistical analysis

The Shapiro-Wilk test was used to determine whether data approximated a normal distribution. To validate the ex-vivo bone-loading model, baseline extensometer motion was subtracted from extensometer motion recorded after slot creation. Because data did not approximate a normal distribution, the Wilcoxon Signed Rank test was used to determine whether data for each slot group differed from a theoretical median of zero (no extensometer displacement). Distribution of condyles between groups was examined using the Chi-squared test. The Wilcoxon Signed Rank test or the Student's *t* test for paired data, as appropriate, were used to analyze pathological change in the lateral and medial joint regions. The correlative relationships between measurements of fatigue crack dimensions on CT images and extensometer micromotion and subjective and objective measures of pathologic change in the joint surface were examined using the Spearman Rank test. Baseline micromotion for bones with naturally occurring fatigue cracks was determined from extensometer measurements of all condyles without fatigue cracks evident on CT examination (n = 14 condyles). A mean baseline extensometer reading was subtracted from the mean micromotion value for each bone before analysis. The Spearman Rank test was also used to determine whether a correlative relationship existed between athletic history (horse age, number of race starts, number of career wins, and racing surface [turf or dirt]) and fatigue crack dimensions measured by CT. A similar analysis was performed for crack micromotion measured by mechanical testing, and subjective and objective measurement of pathologic change.

The relationship between fatigue crack micromotion from mechanical testing and crack dimensions was also examined using logistic regression. Extensometer micromotion was coded as 1 – motion is >15% above baseline, or 0 – ≤15% above baseline for condyles without CT evidence of subchondral fatigue cracks from the pool of bones with naturally occurring fatigue crack arrays. 15% above baseline was arbitrarily chosen to create a conservative assessment of crack micromotion within the statistical model. Horse identity and the presence or absence of a proximal sesamoid bone fracture were included as variables in the logistic regression model. For crack measurement methods with significant results in the logistic regression model, postestimation was used to determine probability of a positive outcome for each CT measurement. Results were considered significant at *p*<0.05. Fatigue cracks with a probability estimate >0.8 for micromotion 15% above baseline were considered clinically relevant.

## Results

### Digital radiography

DR radiographs were made of 76 forelimbs (**Fig. 1**). Fifteen limbs (20%) revealed a fracture of MC3. Eleven limbs (14%) had a condylar fracture (9 lateral and 2 medial). A comminuted diaphyseal fracture was seen in 4 limbs (5%). These limbs were excluded from further analysis (**Fig. 1**). Lesions other than a fracture were identified in distal MC3 bone in 2 of 76 limbs. In one limb, a small radiolucency in the lateral condylar groove was seen, which, subsequently, was associated with a fatigue crack in the subchondral plate identified using CT. Severe POD of both condyles characterized by a subchondral bone defect and associated radiolucency adjacent to the joint surface was seen in another limb. These changes were also visible on CT. Biaxial proximal sesamoid fracture was seen in 12 of 76 limbs (16%) and uniaxial proximal sesamoid fracture was seen in 6 of 76 limbs (8%). Condylar fracture was seen concurrently in three limbs with proximal sesamoid fracture.

Metacarpophalangeal joint osteoarthritis was seen in three limbs, and evidence of rupture of the intersesamoidian ligament (abnormal distance between both proximal sesamoid bones) was seen in one limb. Lateral condylar fracture was also seen concurrently with a comminuted fracture of the proximal phalanx in one limb.

### Computed tomography

**Figure 4:**
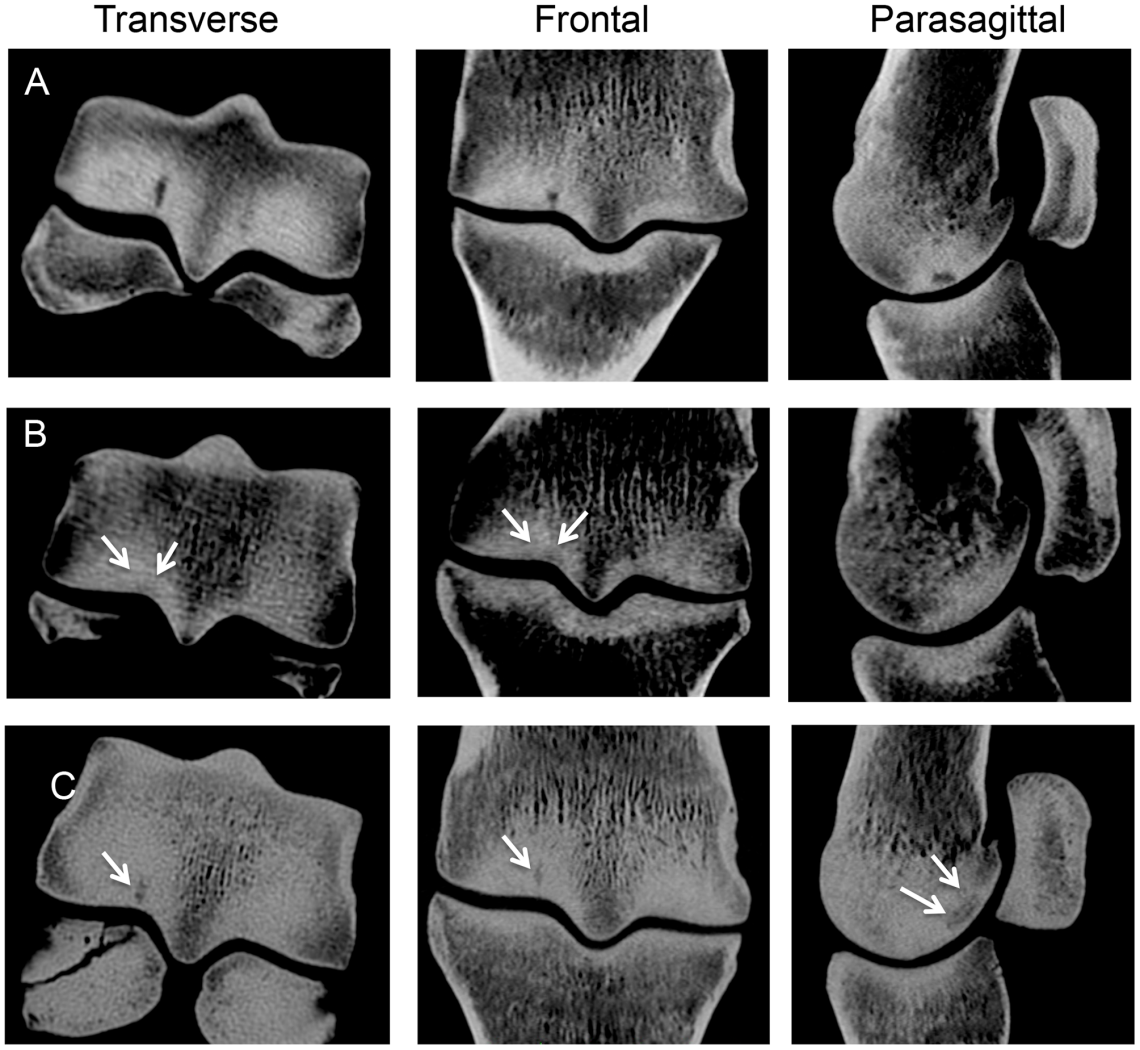
Representative computed tomography images of the distal end of the third metacarpal bone illustrating variation in appearance of parasagittal subchondral crack arrays in the condylar grooves of racing Thoroughbreds. (**A**) A large subchondral crack is evident within the lateral condylar groove. The crack is evident in all three planes. (**B**) In this horse, a small radiolucency is evident in the subchondral plate in the condylar groove (arrows) associated with the presence of a smaller array of fatigue cracks in the transverse and frontal plane images (arrows). (**C**) In this horse, a small well-defined crack is present in the subchondral bone of the medial condylar groove (arrow), which was associated with a large parasagittal crack area that exceeded 30 mm^2^. Fracture of the adjacent proximal sesamoid is also evident.

**Figure 5:**
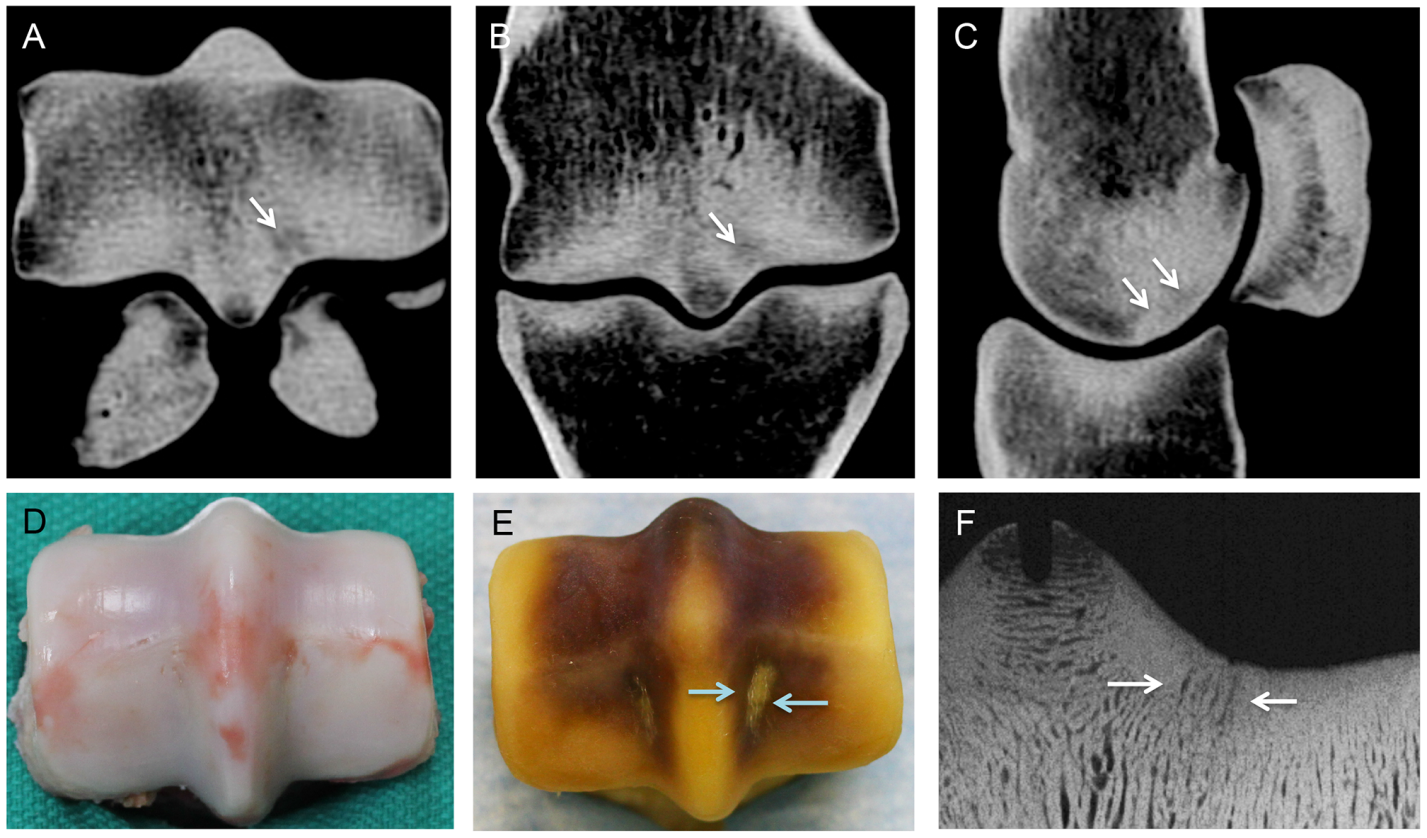
Representative images of the distal end of the third metacarpal bone illustrating the appearance of a large parasagittal subchondral crack array in the lateral condylar groove of the joint surface (arrows) evident on transverse (**A**), frontal (**B**), and parasagittal reconstructed computed tomography images (**C**). A large array of subchondral cracks is present in the subchondral bone of lateral condylar groove (arrows) with relatively little change in the overlying cartilage (**D, E**). An array of fatigue cracks that extend into the proximal part of the subchondral plate is also evident (arrows) on a microradiograph of an oblique frontal bone section (**F**).

**Figure 6:**
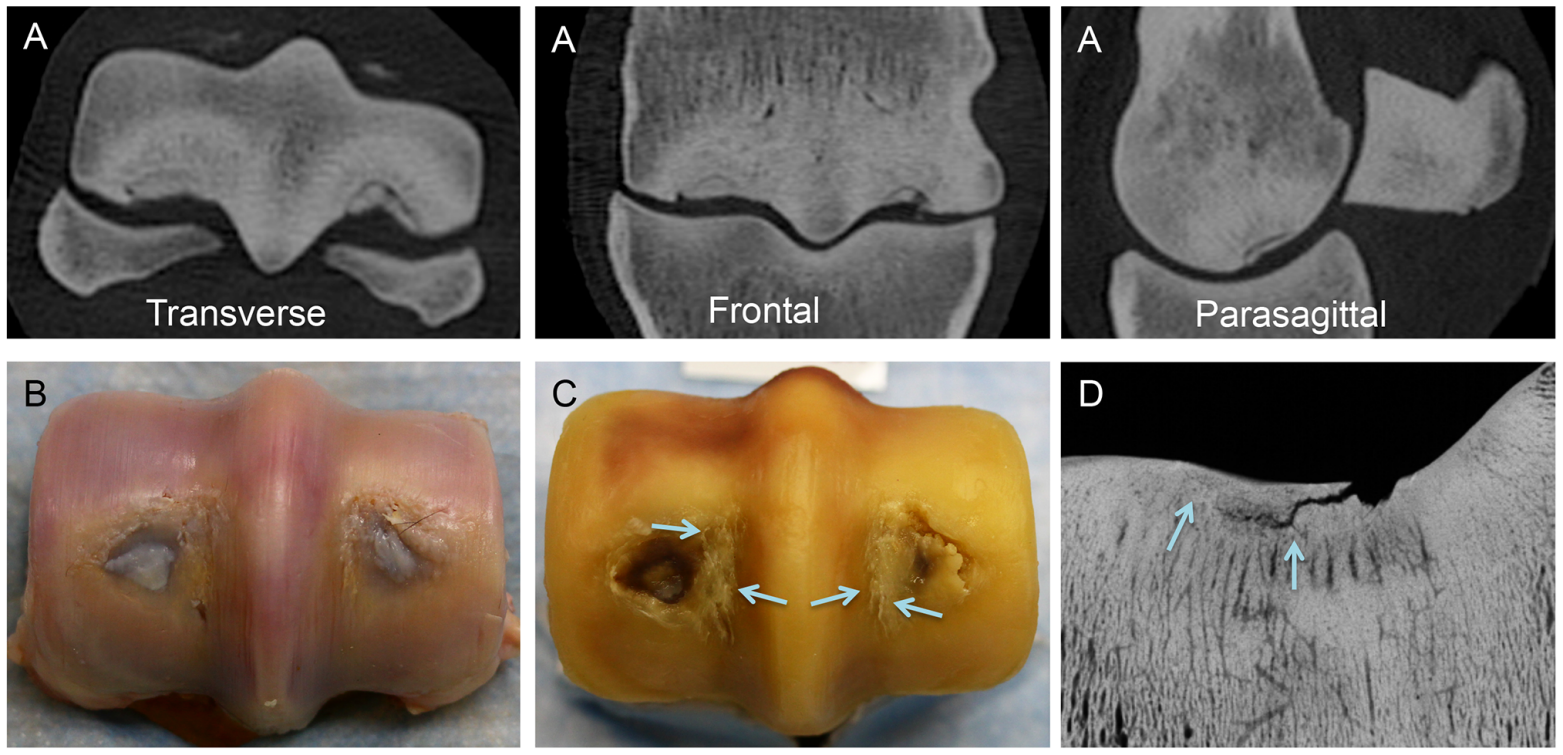
Representative images of the distal end of the third metacarpal bone illustrating articular saucer stress fracture formation in a racing Thoroughbred horse with severe palmar osteochondral disease. (**A**) In reconstructed computed tomography images, a circular articular saucer fracture is present in both condyles. A halo of reduced bone density adjacent to the saucer fracture is caused by the reparative remodeling response in the subchondral plate. Extensive adaptive subchondral sclerosis is also evident. (**B**) Hyaline cartilage overlying the stress fracture has been replaced by fibrocartilage. (**C**) Extensive fatigue damage to the underlying subchondral plate is also evident, including parasagittal cracks in the condylar grooves (arrows). (**D**) Propagation of the fracture line at the interface of the vascular remodeling response to the fatigue injury in the subchondral plate is evident (arrows) on a microradiograph of an oblique frontal bone section.

Sixty-one bones were examined via CT imaging (see Fig. 1 for exclusions). Palmar subchondral bone sclerosis was identified in all condyles studied. Parasagittal crack arrays in the palmar subchondral plate of the distal end of the MC3 bone were identified in 42 of 122 condyles (34%), or 27 of 61 limbs (44%) (Table 1). Of those, 24 were located in a lateral condyle, and 18 in a medial. Fifteen limbs had cracks in both the medial and lateral grooves. The appearance of these subchondral lesions within the condylar grooves varied tremendously in shape and density, ranging from a faint lucency to a distinctive crack in the subchondral plate (Figs. 4, 5). POD was found in 46 of 122 condyles (38%), or 28 of 61 limbs (46%) (Fig. 6). In addition, several other fetlock joint abnormalities were identified, including fractures of the proximal sesamoid bone (15 limbs), osteoarthritis (10 limbs), dorsoproximal proximal phalanx fracture (7 limbs), irregularities on the dorsoproximal aspect of the proximal phalanx (5 limbs), irregularities of the distal mid-sagittal ridge (3 limbs), enthesophytosis of the proximal sesamoid bone (2 limbs), and frontal proximal phalanx fracture (1 limb). Fractures of 26 proximal sesamoid bones were seen. Of the 26 fractures, 12 were comminuted, 7 were mid-body, 5 were basilar, and 2 were apical. Four limbs were not studied further because of the absence of CT lesions (Fig. 1).

**Table 1:** Distribution of fetlock joint abnormalities in Thoroughbred racehorses identified by radiographic imaging and direct observation.

	Digital radiography	Computed tomography	Observation of articular cartilage	Observation of the subchondral plate
	***Wear lines***			
Horses (n = 38)	n/a	n/a	20 (53%)	n/a
Bones (n = 57)	n/a	n/a	24 (42%)	n/a
Condyles (n = 114)	n/a	n/a	46 (40%)	n/a
	***Parasagittal subchondral crack array***			
Horses (n = 38)	1 (3%)	21 (55%)	23 (61%)	25 (66%)
Bones (n = 57)	1 (2%)	27 (47%)	31 (54%)	31 (54%)
Condyles (n = 114)	1 (1%)	42 (37%)	58 (51%)	51 (45%)
	***Palmar osteochondral disease***			
Horses (n = 38)	1 (3%)	19 (50%)	23 (61%)	29 (76%)
Bones (n = 57)	1 (2%)	27 (47%)	32 (56%)	41 (72%)
Condyles (n = 114)	2 (2%)	42 (37%)	53 (46%)	67 (59%)
	***Proximal sesamoid fracture***			
Horses (n = 38)	15 (39%)	15 (39%)	n/a	n/a
Bones (n = 57)	15 (26%)	15 (26%)	n/a	n/a
Condyles (n = 114)	27 (24%)	27 (24%)	n/a	n/a

**Note:** One hundred fourteen condyles from 57 MC3 bones from 38 horses were dissected and digested to evaluate the articular surface (see also Fig. 1). Subchondral plate observations were made after cartilage digestion.

### Visual examination and photography

The distal end of the MC3 bone was examined in 57 limbs, after exclusion for MC3 fracture (15 limbs) and absence of CT lesions (4 limbs) (**Fig. 1**). On gross examination of the joints before digestion of articular cartilage, multiple different pathologies were identified (**Table 1**).

#### Wear lines

Wear lines were identified on the cartilage of the distal end of 24 MC3 bones (42%).

#### Parasagittal subchondral crack arrays

Results are summarized in **Table 2**. Focal or linear cartilage defects were present in the condylar grooves of 58 of 114 condyles (51%). Of these 58 condyles, parasagittal subchondral crack arrays were identified in 33 (57%) after articular cartilage digestion (**Figs. 4** **,** **5**). No cracks in the subchondral plate were seen in the other 25 condyles. After cartilage digestion, subchondral bone crack arrays were identified in 51 of 114 condyles (45%), of which 33 were also identified before cartilage digestion. Of those 51 condyles with visible cracks after removal of the articular cartilage, 28 were evident with CT imaging (55%). Subchondral cracking identified in 11 condyles using CT imaging had no visible lesions in the joint surface of the condylar grooves. Median (range) severity scores for lateral and medial parasagittal linear cartilage defects were 8 (0,48) and 10 (0,42) respectively; severity of disease was not significantly different between lateral and medial condyles. Median (range) severity scores for lateral and medial parasagittal fatigue crack arrays in the subchondral plate were 21.5 (0,92) and 1 (0,90) respectively. Severity of subchondral fatigue damage was increased in the lateral condyle, compared with the medial condyle (*p*<0.05). Severity of subchondral fatigue damage assessed by subjective scoring was not significantly correlated with crack dimensions measured on reconstructed CT images or extensometer micromotion during mechanical testing.

**Table 2:** Identification of parasagittal subchondral crack arrays in the distal end of the third metacarpal bone of Thoroughbred racehorses by computed tomography and direct observation.

	Computed tomography	Observation of articular cartilage	Observation of the subchondral plate
**Computed tomography**	42 (37%)		
**Observation of articular cartilage**	30 (26%)	58 (51%)	
**Observation of the subchondral plate**	28 (25%)	33 (29%)	51 (45%)

**Note:** One hundred fourteen condyles from 57 MC3 bones from 38 different horses were dissected and digested to evaluate the articular surface (see also Fig. 1). Data represent number of condyles. Percentages represent proportion of total (114 condyles).

#### Palmar osteochondral disease

POD was identified in 67 of 114 condyles (59%) after cartilage digestion. Abnormality in the cartilage overlying the subchondral bone lesion was evident in 50 (75%) of these condyles (**Fig. 6** **,** **7**). Of the 67 condyles in which POD was identified after removal of the articular cartilage, 39 were evident with CT imaging (58%). Median (range) severity scores for lateral and medial parasagittal fatigue crack arrays in the subchondral plate were 5 (0,54) and 8 (0,48) respectively. Severity of POD was increased in the medial condyle, compared with the lateral condyle (*p*<0.05).

**Figure 7:**
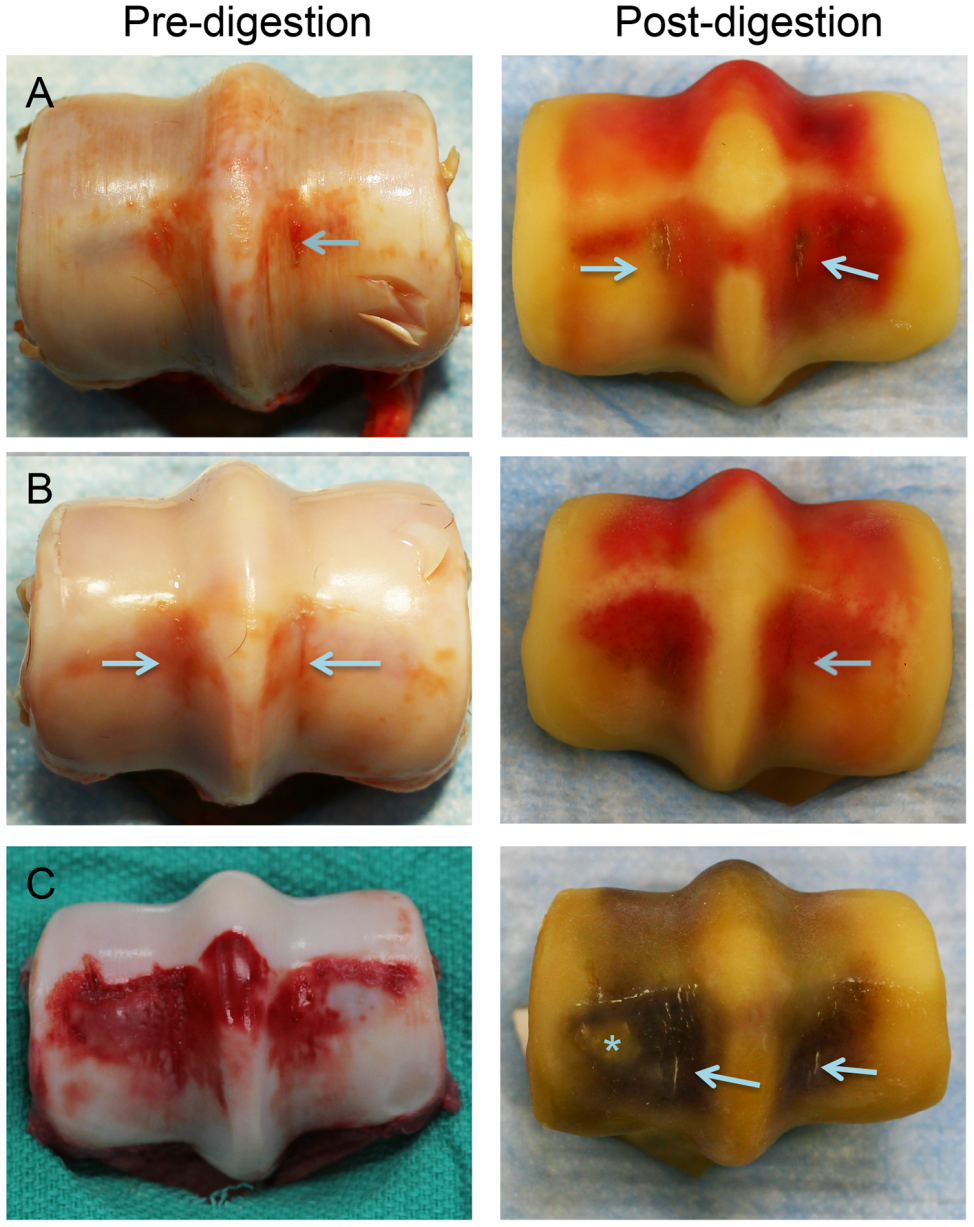
Representative images of the distal end of the third metacarpal bone before and after cartilage digestion illustrating fetlock abnormalities commonly associated with the presence of subchondral fatigue crack arrays. Extensive wear lines in hyaline cartilage (**A**), subchondral bruising (**A–C**) and flattening of the palmar region of the condyles (**C**) were commonly found. In some horses parasagittal defects in the hyaline articular cartilage of the condylar grooves were of mild severity and associated with varying degrees of fatigue cracking of the underlying subchondral plate (arrows) (**A–C**). Palmar osteochondral disease was commonly associated with condylar groove fatigue cracks (* in **C**). Images obtained from the same horses as Figure 4.

### Ex-Vivo Bone Loading Model

In the 3 mm slot group, there were 22 condyles (14 medial and 8 lateral) analyzed from 19 MC3 bones. In the 5 mm slot group, there were 24 condyles (11 medial and 13 lateral) analyzed from 20 MC3 bones. Three condyles did not yield interpretable data and were excluded due to fracture of the bone during biomechanical testing. Six other condyles were excluded because of failure of extensometer data collection (**Fig. 1**).

**Figure 8:**
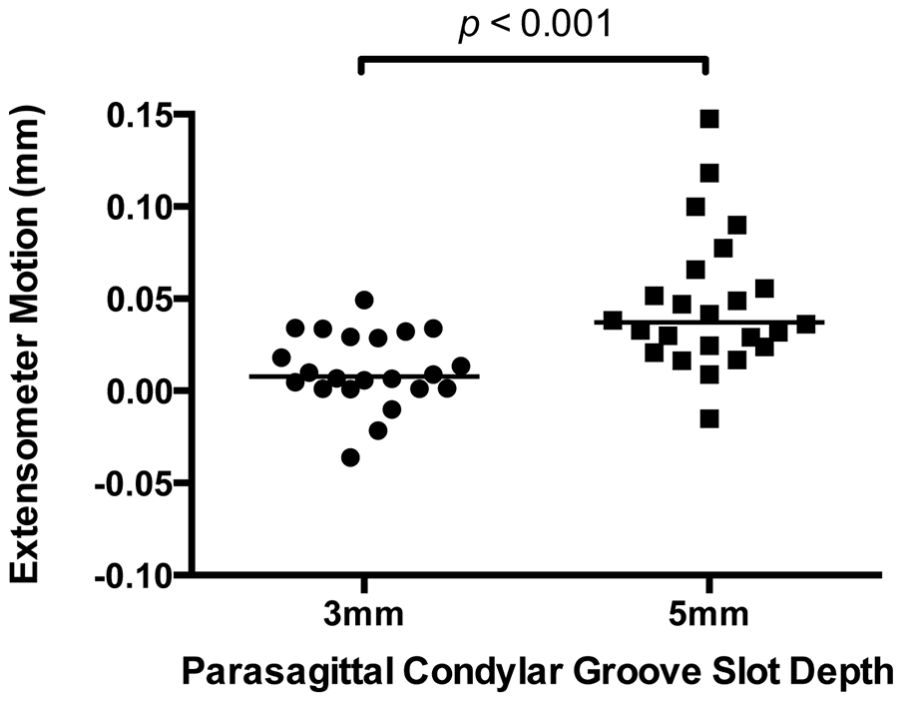
In our ex-vivo biomechanical model, extensometer micromotion associated with creation of shallow parasagittal slots in the palmar region of the condylar groove was used to model naturally occurring arrays of fatigue cracks. In both the 3-corrected extensometer motion was significantly different from zero (no motion) (*p*<0.001). Increased slot depth was associated with increased micromotion. In our model, the condyle was loaded at −7,500 N in compression. In the scatterplot, the horizontal bar represents the median value for each group.

There was no significant difference in the distribution of medial to lateral condyles in both groups. In both the 3 mm and 5 mm groups, significant micromotion was detected with compressive loading, (*p*<0.001), with median values of 0.008 mm and 0.038 mm, respectively. Micromotion was significantly increased in the 5 mm slot group when compared with the 3 mm slot group (*p*<0.001) (**Fig. 8**).

### Relationship between crack micromotion and crack dimensions derived from computed tomographic imaging

MC3 bones from 24 limbs were used for mechanical testing. Of the 48 condyles, 6 did not yield interpretable data and were excluded because the bone sustained a fracture during mechanical testing. Iatrogenic fractures did not propagate from the condylar groove, and affected either the potted region of the bone shaft (n = 2) or the region of the condyle in direct contact with the actuator (n = 4). Four other condyles were excluded because of failure of extensometer data collection. Mechanical data were acquired for the remaining 38 condyles (**Fig. 1**). These condyles were derived from 21 bones from 18 Thoroughbred horses.

**Figure 9:**
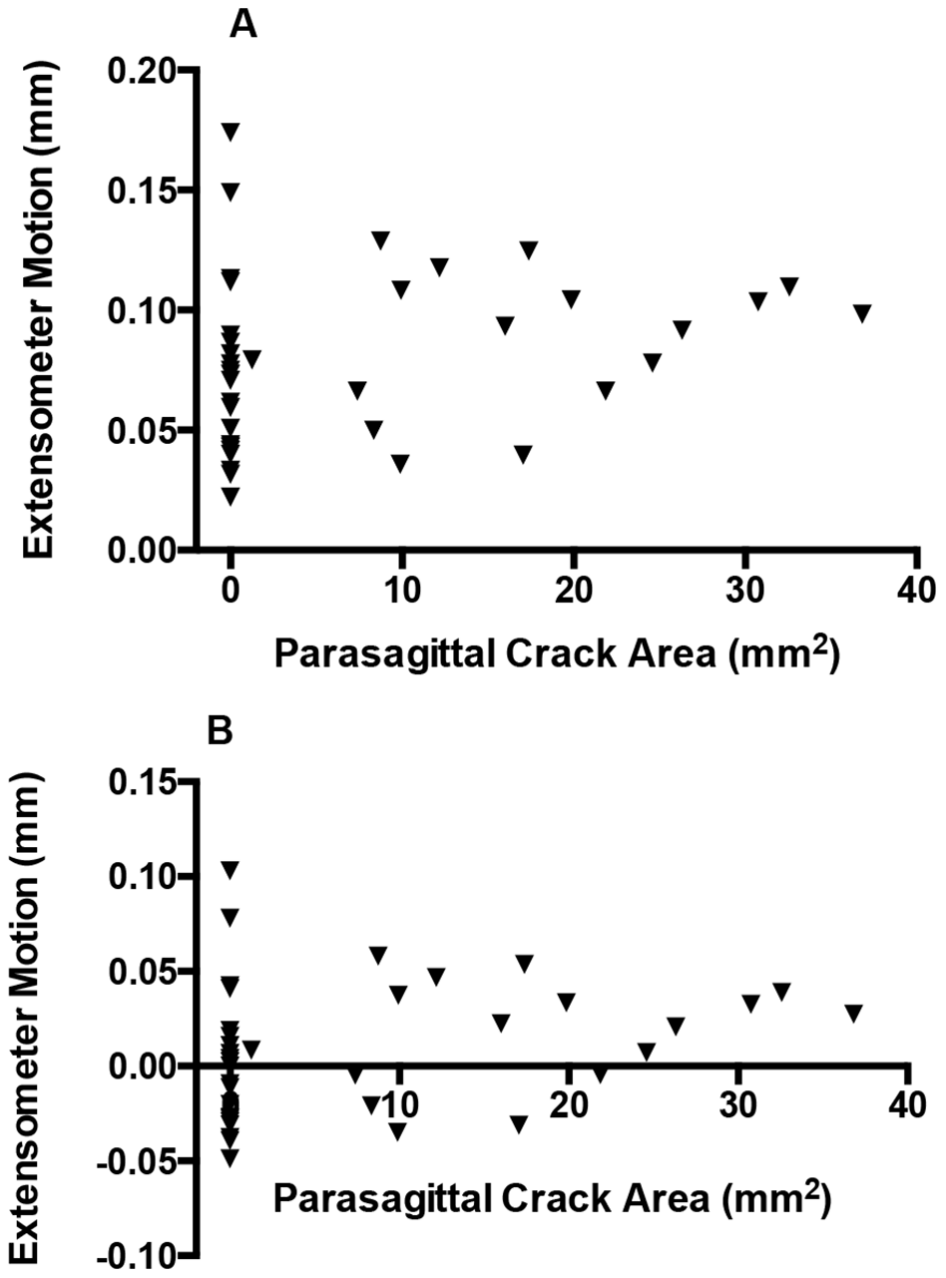
In our ex-vivo biomechanical model, extensometer micromotion associated with naturally occurring parasagittal fatigue cracks in the palmar region of the condylar groove was also determined. (**A**) There was a significant correlation between extensometer motion and parasagittal crack area measured from reconstructed computed tomographic images (S_R_ = 0.32, *p*<0.05). (**B**) Extensometer motion corrected for baseline. Micromotion 15% above baseline was detected in 18 of 38 condyles (47%).

Micromotion 15% above baseline was detected in 18 of 38 condyles (47%) (**Fig. 9**). Median micromotion corrected for the baseline value was 0.007 mm, with a maximum value of 0.103 mm. There was a significant positive correlation between extensometer micromotion and parasagittal crack area measured from reconstructed CT images (S_R_ = 0.32, *p*<0.05). Correlations with transverse and frontal plane crack lengths were not significant. Similarly, correlations with volumetric and surface measurements on 3D reconstructed images were not significant. All CT measurements were significantly correlated with each other (S_R_>0.74, *p*<0.001). When extensometer motion data were transformed into a binary format, we found a significant relationship between crack dimensions on CT and the presence of fatigue crack micromotion (*p*<0.05 for frontal plane crack length, parasagittal crack area, crack volume, and crack surface area, when data were corrected for motion 15% above baseline (**Table 3**); significance was greatest for parasagittal crack area. For parasagittal crack area, a similar result was obtained with a simple baseline correction (*p* = 0.06). Comparisons of other CT measurements with baseline-corrected crack micromotion were not significant. The presence of a proximal sesamoid fracture was not significantly correlated with subchondral crack micromotion and was not a significant factor in the logistic regression model.

**Table 3:** Relationship between computed tomography measurement of parasagittal fatigue crack dimensions and detection of crack micromotion during mechanical loading.

	Statistical Model	Odds ratio	95% Confidence Interval	Likelihood Ratio Chi-square	Significance
**Crack length in the transverse plane**	Crack length + horse			5.57	0.06
	Crack length				0.41
**Crack length in the frontal plane**	Crack length + horse			7.79	0.02
	Crack length				0.1
**Parasagittal crack area**	Crack area + horse			9.53	0.01
	Crack area	1.08	1.00–1.16		0.05
**Crack volume**	Crack volume + horse			6.90	0.03
	Crack volume				0.17
**Crack surface**	Crack surface + horse			7.43	0.02
	Crack surface				0.13

**Note:** A logistic regression model was used to determine whether there was a significant relationship between fatigue crack dimensions determined by measurement from computed tomography images and crack micromotion under clinically relevant joint loads in an ex-vivo model (micromotion corrected to 15% above baseline) (n = 38 condyles from 21 bones from 18 horses). Within the multivariate models of CT measurement parameter and horse identity, parasagittal crack area was the only CT measurement that yielded a significant result.

In the logistic regression model, 3 of 38 condyles (7.9%) in the limbs of three different horses had parasagittal fatigue cracks in the condylar groove with a parasagittal crack area measurement that exceeded 30 mm^2^. Specimens with parasagittal crack area measurements above this value had a probability of >0.8 that crack micromotion in our biomechanical model would exceed baseline +15% and be deemed clinically significant (**Figs. 4** **,** **5** **,** **7**).

### Subchondral fatigue crack histomorphometry

Mean ± standard deviation N.Cr/B.Bd was 0.62±0.34 and 0.55±0.32 respectively for the lateral and medial condyles; severity of histologic fatigue damage was not significantly different between lateral and medial condyles. Severity of histologic fatigue damage was not significantly correlated with crack dimensions measured on reconstructed CT images or extensometer micromotion during mechanical testing. Examination of the oblique frontal bone sections after mechanical loading did not reveal any evidence of crack extension from mechanical testing. One or two unstained cracks were identified in the articular surface of the condylar groove in 6 of 38 condyles after mechanical testing.

### Relationship between athletic history and severity of subchondral fatigue damage

Mechanical data were obtained for 38 condyles from 22 limbs of 18 horses. Of these 18 Thoroughbreds, a racing history was available for 15 animals. The study group was comprised of 7 females and 8 males consisting of 5 stallions and 3 geldings. The median age was 4 years old (range of 2 to 8). Median number of career starts was 12 (range of 0 to 46). Seven horses were racing on dirt and 4 on turf. One horse never raced. Athletic history was not available for the other three horses. There were no significant correlations between athletic history and the magnitude of parasagittal fatigue crack dimensions from CT imaging of the distal end of the MC3 bone. Similarly, there were no significant correlations between athletic history and detection of crack micromotion. Subjective scoring and histomorphometric assessment of parasagittal subchondral fatigue damage, but not severity of POD, was significantly correlated with horse age and athletic activity (**Table 4**). Significant correlations between severity of subchondral fatigue damage and racing surface were not detected.

**Table 4:** Relationship between athletic history and severity of pathologic change observed in the joint surface of the distal end of the third metacarpal of racing Thoroughbreds.

Parameter	S_R_	Significance
***Horse age***		
Condylar groove cartilage defect severity score	−0.25	NS
Condylar groove subchondral bone damage severity score	0.61	*p*<0.001
N.Cr/B.Bd, #/mm	0.57	*p*<0.001
***Number of race starts***		
Condylar groove cartilage defect severity score	−0.38	*p*<0.05
Condylar groove subchondral bone damage severity score	0.50	*p*<0.005
N.Cr/B.Bd, #/mm	0.37	*p*<0.05
***Number of career wins***		
Condylar groove cartilage defect severity score	−0.30	NS (*p* = 0.09)
Condylar groove subchondral bone damage severity score	0.35	*p* = 0.05
N.Cr/B.Bd, #/mm	0.37	*p*<0.05

**Note:** Correlations with severity scoring of palmar osteochondral disease were not significant for any athletic history parameter (n = 32 condyles).

## Discussion

Collectively, past observations suggest that condylar stress fractures arise from site-specific accumulation of fatigue damage in the subchondral plate of the distal end of the MC3/MT3 bone [12]–[16]. However, the mechanism by which fatigue damage ultimately precipitates structural failure of the MC3/MT3 bone during athletic activity is poorly understood and little investigated. Results from our ex-vivo model suggest that it is likely that some degree of fatigue crack micromotion is present in a large proportion of Thoroughbred racehorses during training and racing. Although subchondral crack micromotion may not be the only determinant of a stress fracture, microcrack propagation, as opposed to initiation, is likely an important component of stress fracture development [29]. Microstructural features of the subchondral plate, such as resorption spaces, may also have an important role in crack propagation [14], [29]. Our results suggest that CT imaging could be used to quantify subchondral fatigue crack dimensions in racing Thoroughbred horses and assess risk of condylar fracture in clinical longitudinal studies. Horses with parasagittal crack arrays that exceed 30 mm^2^ may have a high risk for development of condylar fracture; based on our results it is likely that fatigue crack arrays exceeding these dimensions are exposed to micromotion during high speed running and are, therefore, vulnerable to catastrophic crack propagation [14], [15].

During initial screening with DR, we found that condylar fracture was present in 14% of limbs of the Thoroughbred horses in this study. This observation is similar to other epidemiological studies that suggest that condylar stress fracture is commonly associated with catastrophic injuring in racing Thoroughbreds [5]–[7]. Fractures of the proximal sesamoid bones were also commonly detected using DR and CT imaging in the Thoroughbred horses of the present study. The majority of these were comminuted. Similar observations have been made in clinical studies in Thoroughbred racehorses with catastrophic injuries [6], [7].

The lesions we identified on examination of the articular surface before and after cartilage digestion were similar to earlier studies [11], [12], [23], [30] and support the concept that pathological changes to the fetlock joint are common in racing Thoroughbreds, including young horses at the beginning of their racing career.

Previous work from our laboratory suggests that cross-sectional imaging, particularly with CT, is superior to digital radiography for lesion identification in the fetlock joint of racing Thoroughbreds [22], [23]. This observation was reaffirmed in the present study. However, CT imaging was only able to identify 55% of condyles with visible subchondral cracking after cartilage digestion. This might be explained by the fact that some visible crack arrays are small and only affect the subchondral bone plate superficially, thus preventing identification on CT examination. Histologic examination of the distal joint surface of the MC3 bone from racing Thoroughbreds suggests that fatigue injury is initiated in the calcified cartilage of the joint surface [14]. However, in the present study, we found that subchondral cracking identified in 11 condyles on CT imaging had no visible lesions in the joint surface of the condylar grooves after cartilage digestion. This may suggest site-specific remodeling in the subchondral plate proximal to the articular surface is also an early event in the disease mechanism. Alternatively, crack initiation may occur deeper within the subchondral plate in some horses. It should be noted that our evaluation of these condyles occurred at a specific point in time, and cannot consider how adaptive remodeling may lead to changes in pathological features within the subchondral plate over time. Therefore, it is possible that some of these lesions represent the early phase of the damage process before development of a more extensive crack array in the subchondral plate, or alternatively the beginning of a healing process.

The appearance of parasagittal subchondral crack arrays in the condylar grooves of the MC3 joint surface was quite variable using CT imaging. Subchondral cracks surrounded by radiolucency may have a greater area or volume than cracks with more clearly defined margins. This is likely important, as stressed volume is thought to be an important factor in the pathogenesis of stress fracture [31]. Our regression analyses suggested that measurement of parasagittal crack area, as opposed to transverse or frontal plane measurements of crack length, yielded the best predictor of crack micromotion. Detection of radiolucency in the parasagittal condylar groove on CT may suggest that the array of interconnected fatigue cracks occupies a larger volume in the subchondral plate. This concept could be examined by more detailed histomorphometric examination of fatigue-damaged bone with this CT abnormality. In the present study, we did not identify a significant correlation between crack array dimensions on CT and objective measurement of histologic fatigue damage or subjective grading of joint pathology. Even though CT imaging was performed at high resolution, precise determination of crack margins was not always straightforward. Improvements in image resolution may help to identify horses with more subtle fatigue damage in the subchondral plate of the distal end of the MC3/MT3 bone.

CT imaging was also able to identify POD in a large proportion of the condyles studied. However, not all POD lesions seen on examination of the subchondral plate after cartilage digestion were evident with CT imaging. The main reason for this discrepancy is that those POD lesions not identified on CT imaging were mostly a discoloration of the palmar condyle area, and not a bony defect. Ten of 24 limbs in the naturally occurring crack group were affected by POD, potentially complicating measurement of subchondral crack motion during mechanical testing. In specimens with this lesion, care was taken to place the extensometer needle axial to the POD lesion. By doing so, any POD-associated micromotion should not be included in the motion measured by the extensometer. Although subchondral bone cracks and POD are different clinical entities, they have a similar etiology and both represent fatigue injury to the joint surface [16]. They are a consequence of repetitive stresses from strenuous exercise [12]–[14], [16], [26], [27], which predisposes them to occurring simultaneously in the same condyle. It is unclear why some horses develop POD instead of parasagittal subchondral fatigue cracks, or vice versa.

To validate our ex-vivo model, we measured micromotion before and after creation of parasagittal slots of two different depths in the palmar region of the condylar groove. Data from this initial experiment supported the validity of our biomechanical model and yielded micromotion values that approximated the values obtained from bones with naturally occurring fatigue crack arrays. Loading conditions for the model were based on earlier studies [20]. Although removal of the articular cartilage may have influenced load distribution during testing, cartilage removal was necessary to observe parasagittal subchondral crack arrays, if present.

We found evidence of fatigue crack micromotion in 47% of condyles, suggesting that a large proportion of racing Thoroughbreds may experience some degree of crack micromotion in vivo. This is an important observation, since it is unlikely that articular fatigue damage will heal well clinically in the face of ongoing instability from habitual training and racing. Horses with parasagittal crack areas that exceed 30 mm^2^ are likely to experience substantial crack micromotion under high joint loads, based on our regression analysis. This result was identified in 7.9% of horses, suggesting that a large number of Thoroughbreds racing in the United States are vulnerable to condylar stress fracture during racing.

In this experiment, extensometer motion was detected in condyles without detectable subchondral crack arrays. This is likely a consequence of the large compressive load that was applied to the joint surface during testing to model weight-bearing during high-speed running. Rotational motion and creep of some of the bones inside the potting cylinder was noticed during the mechanical testing and there was a tendency for some degree of extensometer drift during the five loading cycles of each test. To account for this limitation, mechanical data were baseline-corrected before statistical analysis. Further, we chose to only consider crack micromotion as relevant when it increased ≥15% above baseline. As there is no established model to determine what amount of motion is truly relevant, this value was chosen arbitrarily as a conservative approach to data interpretation. Development of an improved method for measurement of crack motion during mechanical testing may be help improve precision of mechanical testing in future work.

Histomorphometric quantification and severity scoring of subchondral cracking were significantly correlated with horse age and number of race starts, suggesting that accumulation of MC3 subchondral fatigue injury is related to habitual athletic activity. Cumulative cyclic loading of the fetlock is influenced by accumulation of racing starts as well as high-speed timed workout. Another important component of fetlock loading history is the frequency of these events, which can significantly influence risk of a catastrophic musculoskeletal injury [32]. Severity of the defect in the condylar groove cartilage was significantly and inversely related to number of race starts suggesting that cartilage damage is an early feature of joint injury from habitual athleticism. We did not identify any significant correlation between the cumulative racing history and the magnitude of parasagittal fatigue crack dimensions from CT imaging of the distal end of the MC3 bone, or detection of crack micromotion. However, data were only collected from a limited number of horses, so this finding should be interpreted with caution in the absence of relevant data describing the training activity, as the amount and intensity of workout sessions is likely important to accumulation of subchondral fatigue damage over time. More work is needed to comprehensively understand the relationship between athletic history and subchondral fatigue injury. Clinically, condylar fractures are more common in younger horses that may not have an extensive racing history [1], [3], [25].

A limitation of this project in accurately modeling an *in vivo* scenario was our use of frozen limbs collected from catastrophically injured racing Thoroughbreds. Limbs were thawed for DR and CT imaging and then refrozen until further dissection, digestion, and basic fuchsin staining. It is possible that post-mortem change and freeze-thaw effects could have influenced our results. Such effects are likely relatively small in a high load biomechanical model. Furthermore, all specimens tested were treated in a similar manner. Removal of the articular cartilage was necessary to appreciate the subchondral crack array in the condylar grooves. Another limitation is that we studied an isolated single bone model with our mechanical testing. Therefore, our study does not address the role of the first phalanx in loading of the distal MC3 bone. It is likely that the articulation between the proximal phalanx and the metacarpus and the loading pattern resulting from this is of importance in the pathogenesis of condylar fracture. Although currently unknown, this interaction, as well as the forces placed on the condyles by the adjacent soft tissues structures (collateral ligaments) probably plays a role in the formation and propagation of these fractures. Our goal was to isolate one portion of the bone that is of known importance in condylar fractures to estimate crack micromotion during direct loading via the articulation between the condyle and the sesamoid. While of great importance, addressing the roles of other anatomical structures is outside the scope of this study, but relevant to future work. The presence of a proximal sesamoid fracture was not significantly related to detection of crack micromotion in our model. Although actuator placement modeled the position of the proximal sesamoid during mechanical testing, in-vivo loading of the distal MC3 bone by the proximal sesamoid may be different. This may explain the iatrogenic fracture that occurred in some bones during testing. Finally, athletic history was obtained from a relatively limited number of horses. Additional epidemiological work is needed to comprehensively understand the relationship between habitual athleticism and fetlock injuries, such as accumulation of MC3 subchondral fatigue damage.

The current design of equine CT scanners requires general anesthesia, which limits the use of CT as a diagnostic modality for equine athletes in race training. However, CT scanning of the foot in the standing horse using a gantry in a horizontal orientation has been performed [33]. Results of the present study suggest that this approach to equine CT imaging needs to be expanded to include the fetlock. With cross-sectional imaging of the fetlock, particularly if available as a standing modality, longitudinal screening of a population of Thoroughbred racehorses in training or racing could improve our ability to recognize and monitor the progression of specific pathologic features in vivo. This would be extremely informative to understanding and treatment of distal MC3/MT3 injury or disease. While reduction in joint loading may contribute to bone healing, it is unknown whether healing of condylar groove subchondral cracks occurs if habitual athletic activity is withdrawn.

In conclusion, condylar stress fractures are common in Thoroughbred racehorses. Parasagittal arrays of fatigue cracks in the subchondral plate of the condylar grooves of the distal end of the MC3 bone are readily detectable by CT. Our results suggest that measurement of crack area on reconstructed parasagittal CT images is a predictive marker for crack micromotion at joint loads associated with high-speed running. Consequently, this parameter may prove to be a useful predictive marker for condylar fracture risk in longitudinal in-vivo studies of Thoroughbred racehorses. Improved determination of fracture risk promises reduction in serious injuries during racing and better clinical management of horses with incipient condylar fracture. Furthermore, such knowledge promises improved understanding of the epidemiological relationship between habitual athletic activity and fetlock breakdown injury.
